# Predictors of acute kidney injury after percutaneous nephrolithotomy in adult patients: prospective observational study

**DOI:** 10.1007/s11255-024-03960-7

**Published:** 2024-01-30

**Authors:** Ahmed Mahmoud Hasan, Ahmed Mahmoud Riyad, Mostafa AbdelRazek Ahmed

**Affiliations:** 1 Urology Department, Faculty of Medicine, South Valley University, Qena, Egypt; 2 Urology Department, Faculty of Medicine, Sohag University, Sohag, Egypt

**Keywords:** Acute kidney injury, Percutaneous nephrolithotomy, Nephrolithiasis, Renal function

## Abstract

**Purpose:**

To assess the frequency and the predictive factors of Acute Kidney injury (AKI) in patients undergoing percutaneous nephrolithotomy (PNL).

**Methods:**

A prospective observational work. Demographic, preoperative laboratory data, stone characteristics, and intraoperative and postoperative data were gathered. Perioperative AKI had been defined as an elevation in serum creatinine by ≥ 0.3 mg/dl within 48 h, or ≥ 1.5 times baseline, or urine volume less than 0.5 ml/ kg/hour for 6 hours. A multivariate logistic regression analysis was performed to determine the predictive factors of AKI. ROC curves were utilized to determine the cutoff values of the risk variables. P-values were deemed statistically significant when they were less than 0.05.

**Results:**

A total of 418 participants had been involved. The frequency of AKI was 13.9, and 17.2% of patients with AKI developed CKD. The risk factors were age > 46.5 years, smoking, BMI > 28.5 kg/m^2^, hypertension, diabetes, utilization of angiotensin-converting enzyme inhibitors (ACEI), haemoglobin < 10.8 gm/dl, baseline creatinine > 1.41 mg/dl, eGFR < 65.2 ml/min./1.73 m^2^, serum uric acid > 5.2 mg/dl, stone volume > 1748 mm^3^, large tract size, long operative time, and intra-operative bleeding. Patients with AKI had a notably extended duration of hospitalization (3.2 days ± 0.45 vs 2.1 ± 0.42, p < 0.001).

**Conclusions:**

Perioperative AKI occurred in 13.9% of individuals undergoing PNL. Identification and optimization of the risk factors and meticulous technique during PNL procedures should be attempted to decrease the risk of AKI.

## Introduction

Percutaneous nephrolithotomy (PNL) is the preferred therapy for large, intricate, and numerous renal stones, as well as smaller ones that do not respond to extracorporeal-shock waves lithotripsy (ESWL) [1, 2]. PNL Causes direct renal injury by puncturing the renal parenchyma. Also, stones-associated obstruction or infection may affect kidney function, so urologic patients are considered at high risk for acute kidney injury (AKI) [3].

According to Kidney Disease Improving Global Outcomes (KDIGO), AKI is diagnosed based on the presence of one of the followings: an elevation in the serum creatinine ≥ 0.3 mg/dl within 48 h, an elevation in the serum creatinine≥1.5 times more than the basal creatinine level within the proceeding 1 week or urine output≤0.5 ml/kg/h for 6 hours [4, 5].

The pathogenesis of AKI involves multiple interactions between tubular, vascular and inflammatory factors due to injuries caused by ischemia and/or toxins. This leads to endothelial damage, vasoconstriction, and the activation of inflammatory mediators of the immune system [6]. High-pressure irrigation sometimes used during PNL to gain good vision causes pyelorenal backflow, sepsis and renal damage which are directly related to increased intrarenal pressure [7].

AKI is a significant consequence after surgery in urologic patients, with an incidence ranging from 6.7 to 38.2% [8, 9]. It can lead to poor postoperative complications with, sometimes, the need for intensive care unit admission or dialysis with increased morbidity, longer hospital stay, and more cost [10, 11].

The incidence, risk factors, and long-term complications of AKI after PNL are still poorly defined. There is a scarcity of research that documents alterations in the renal function following PNL [12, 13]; hence, defining the risk factors for AKI following PNL is crucial to avoid and minimize its incidence.

### Patients and methods

This prospective observational study was carried out on individuals who attended our clinics with renal stones who were amenable to PNL and fulfilled the inclusion criteria from March 2019 to March 2022. The institutional ethics committee approval (approval code: SVU-MED-URO0016-4-22-5-403) was obtained. Each participant provided informed written consent.

### Inclusion criteria

All adult patients (age ≥ 18 years) with unilateral renal calculi who were candidates for percutaneous nephrolithotomy with normal contralateral kidneys were included.

### Exclusion criteria

Individuals < 18 years, with bilateral renal stones, solitary functioning kidney, active urinary tract infection, bleeding disorders, compromised renal function with serum creatinine < 2 mg/dl (to avoid complications of PNL which are related to high preoperative serum creatinine), urinary tract malignancies and pregnant females had been excluded.

Demographic data that includes age, sex, smoking habits, body mass index (BMI), chronic diseases including hypertension and DM, drug history and preoperative investigations including preoperative assessment of haemoglobin percentage (Hb%), Total leucocytic count (TLC), hematocrit value (HCT%), serum creatinine, estimated glomerular filtration rate (eGFR) utilizing the CKD-EPI (Chronic Kidney Disease Epidemiology Collaboration) Eq. (2021 update) [14], prothrombin time and concentration, serum uric acid, urine culture (we ensured that all cases had negative urine culture before operation), abdominal ultrasound, non-contrast computed tomography with the assessment of the characteristics of the stones [site, volume of stone in cubic millimetres (stone volume = L × W × D × π × 0.167), and density] were collected.

The intraoperative data including the puncture site, tract size, number of punctures, the demand for blood transfusion, operative time, use of any nephrotoxic drugs, stone clearance by intraoperative fluoroscopy, and any reported intraoperative complications, especially bleeding and hypotension (defined as a mean arterial blood pressure of > 70 mm Hg for < 5 min) were assessed.

### Surgical technique

Preoperative 3rd generation cephalosporins were given to all participants. Under general anaesthesia, the patient was positioned in a lithotomy position, and a 5-French ureteral catheter was placed utilizing a 22-French cystoscope. The position of the ureteral catheter was verified utilizing fluoroscopy followed by positioning the patient in the prone postition; a renal puncture by Chiba needle into the desired calyx was done, followed by insertion of the guide-wire, the track was dilated using serial metallic Alken dilators followed by insertion of the Amplatz sheath. The irrigation fluids utilized throughout the procedure had been prewarmed to the body's temperature in the operating room. Pneumatic lithotripsy, utilizing Swiss lithoclast **®**, was done, a nephrostomy tube was left after completion of the procedure, and the duration of the operation was determined from the moment the skin was first punctured until it was closed.

Postoperative laboratory investigations involved TLC, Hb, HCT % and serial serum creatinine at 12, 24 and 48 h were reassessed, and all standard blood samples were collected at 08:00 h. Calculation of 48 h of urine output was done for all cases. All patients were advised to maintain sufficient hydration by drinking 3.7 L (15.5 cups) of fluids per day in males and 2.7 L (11.5 cups) of fluids per day in females as advised by the U.S. National Academics of Sciences, Engineering and Medicine. The nephrostomy tube had been removed on the subsequent day after the surgery and the ureteric catheter was removed 2 days after removal of the nephrostomy tube.

The assessment of postoperative complications was conducted with the modified Clavien–Dindo grading scale which consists of 5 grades:

1. Grade 1: Any deviation from the normal postoperative course with no surgical treatment, endoscopic or radiological interventions were required. Acceptable therapeutic regimens are drugs such as anti-emetics, antipyretics, analgesics, and diuretics.
2. Grade 2: Pharmacological management other than grade 1. Blood transfusion and total parenteral nutrition are also included.
3. Grade 3: Complications that require intervention. Grade 3 is subdivided into:
  - Grade IIIa: Complications that require an intervention performed under local anaesthesia.
  - Grade IIIb: Complications that require an intervention performed under general or epidural anaesthesia.
4. Grade IV: Life threatening complications requiring ICU admission. Grade 4 is subdivided into:
  - Grade IVa: Single organ dysfunction (including dialysis).
  - Grade IVb: Multiple organ dysfunction.
5. Grade V: Death of the patient.

Postoperative abdominal ultrasound and KUB were done for all patients before discharge to document stone clearance or the presence of any residual stones. Participants who had AKI after surgery were monitored by regularly measuring their creatinine levels and eGFR at 1, 3, 6, and 12 months. According to KDIGO, Chronic kidney disease (CKD) was defined by persistently reduced eGFR below 60 mL/min/1.73 m2, or persistent albuminuria [albumin–creatinine ratio {ACR} ≥ 30 mg/g {≥ 3 mg/mmol}], or both [15]. In this study, CKD was defined by an eGFR below 60 mL/min/1.73 m2 at the end of our follow-up period (one year).

Statistical analysis was done utilizing the SPSS version 24. Categorial variables were displayed as frequencies and percentages [*n* (%)] and were compared using the Chi-square test. Quantitative parameters were displayed as the median and interquartile range (IQR) and were contrasted utilizing Mann–Whitney U test (MW) (as the data was not normally distributed). Univariate linear regression analysis and multivariate logistic regression analysis were utilized to identify AKI risk factors. Receiver operating characteristic (ROC) Curves were utilized to detect the cutoff value, sensitivity, specificity, positive predictive value (PPV) and negative predictive value (NPV) of the studied risk factors of AKI. *P*-values were deemed statistically significant at *p* < 0.05.

### Aim of the study

The objectives of this study is to assess the incidence of postoperative AKI and to determine the independent risk factors forecasting its possible occurrence in patients undergoing PNL.

## Results

**Fig. 1:**
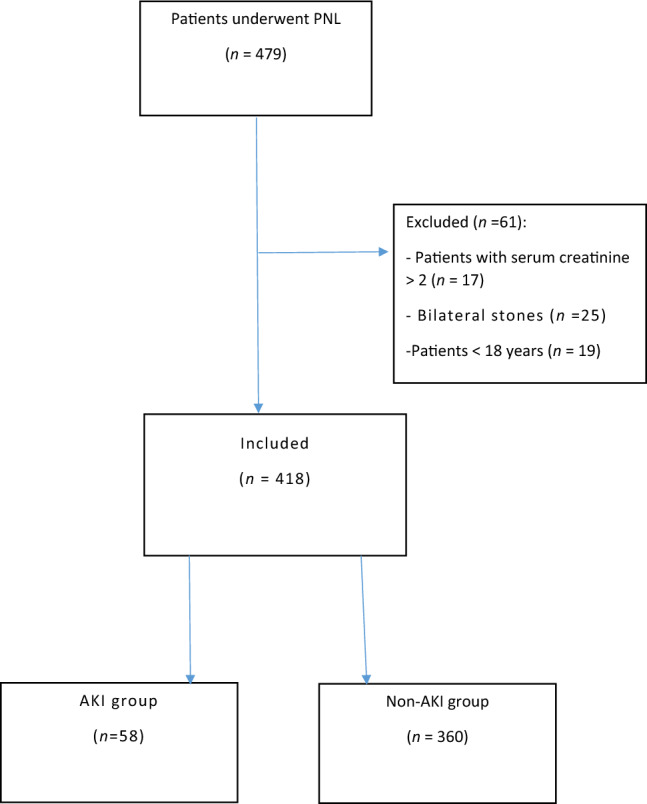
Study flow

In this work, we assessed 479 individuals for eligibility. After exclusions, 418 patients were enrolled. Patients were allocated into 2 groups: group 1 included fifty-eight patients (13.9%) in whom AKI occurred, and group 2 included three hundred and sixty patients (86.1%) without AKI (non-AKI) (Fig.1).

Details of the demographics of the patients and stone characteristics were collected. Statistically significant variations were found among both groups regarding age [54 (47–58) vs 36 (29–45); *p* < 0.001], smoking status, BMI, presence of DM and hypertension, utilization of ACEI, preoperative Hb, creatinine, eGFR, and serum uric acid. No statistically significant variations existed regarding the characteristics of the stones except for the stone burden, which was more in the AKI group (*p* < 0.001) (Table 1).

**Table 1:** Comparisons between AKI and non-AKI patients regarding the preoperative demographic, laboratory, and stone data

		All patients (*n* = 418)		AKI (*n* = 58)		Non-AKI (*n* = 360)				MW/X^2^	*p*-value
Age (years)		40 (30–48)		54 (47–58)		36 (29–45)				**734.5**	** < 0.001**
Gender	Male	250	59.8%	40	69%	210	58.3%			1.17	0.278
	Female	168	40.2%	18	31%	150	41.7%				
Smoking		116	27.8%	38	65.5%	78	21.7%			23.9	** < 0.001**
BMI		26 (24–28)		32 (28.5–32)		25 (24–27.7)				**580.5**	** < 0.001**
Diabetes		32	7.7%	30	51.7%	2		0.6%		**92.5**	** < 0.001**
Hypertension		44	10.5%	30	51.7%	14		3.9%		**60.6**	** < 0.001**
ACEI		28	6.7%	24	41.4%	4		1.1%		**64.8**	** < 0.001**
Hb		12.6 (11.8–13.4)		11.8 (10.7–13.2)		12.7 (11.9–13.4)				**1690.5**	**0.002**
TLC		8 (7–8)		8 (7.5–8.75)		8 (7–8)				2145	0.113
HCT		42 (40–44)		42 (40–44)		43 (40–44.7)				2243.5	0.222
Creatinine		1.1 (0.94–1.2)		1.52 (1.3–1.6)		1.01 (0.91–1.18)				**339**	** < 0.001**
eGFR		78.4 (65.5–97.3)		59.6 (56.5–69.5)		82.8 (70.8–100.9)				**406.5**	** < 0.001**
Uric acid		4.2 (3.8–5)		9.3 (5.9–10)		4.05 (3.6–5)				**428**	** < 0.001**
Stone site	Pelvis	344	82.3%	46	79.3%	298			82%	4.6	0.06
	Upper calyx	16	3.8%	4	6.9%	12			3.3%	0.86	0.353
	Middle calyx	92	22%	18	31%	74			20.6%	1.59	0.206
	Lower calyx	92	22%	10	17.2%	82			22.8%	0.44	0.504
Stone volume		1700 (1420–2040)		2000 (1710–2485)		1672 (1400–2000)				**1425.5**	** < 0.001**
Stone density		1000 (780–1200)		985 (796.2–1200)		1000 (780–1200)				2498.5	0.942

Bold indicates statistically significant difference

X^2^: Chi-square test MW: Mann–Whitney test

Regarding the intraoperative data, statistically significant variations were found among both groups regarding tract size (*p* = 0.003), operative time [80 (70–94) vs 68.5 (64–74) minutes; *p* < 0.001), need for intraoperative blood transfusion (37.9% vs 0.6%; *p* < 0.001), occurrence of intraoperative bleeding and hypotension (24.1% vs 0.6% and 20.7% vs 0% correspondingly; *p* < 0.001). No statistically substantial variations existed regarding the puncture site and the number of punctures (Table 2).

**Table 2:** Comparisons between AKI and non-AKI patients regarding the intraoperative data

		All patients (*n* = 418)		AKI (*n* = 58)		Non-AKI (*n* = 360)		MW/X^2^	*p*-value
Puncture site	**L. Calyx**	152	36.4%	20	34.5%	132	36.7%	0.22	0.894
	**M. Calyx**	264	63.2%	38	65.5%	226	62.8%		
	**U. calyx**	2	0.5%	0	0%	2	0.6		
Puncture No	**1 punc**	410	98.1%	56	96.6%	354	98.3%	0.42	0.516
	**2 punc**	8	1.9%	2	3.4%	6	1.7%		
Tract size		30 (26–30)		30 (30–30)		30 (26–30)		**1931.5**	**0.003**
Operative time		70 (64.5–75)		80 (70–94)		68.5 (64–74)		**1037.5**	** < 0.001**
Blood transfusion		24	5.7%	22	37.9%	2	0.6%	**64.5**	** < 0.001**
Op comp	**Bleeding**	16	3.8%	14	24.1%	2	0.6%	**37.7%**	** < 0.001**
	**hypotension**	12	2.9%	12	20.7%	0	0%	**38.3**	** < 0.001**

Bold indicates statistically significant difference

X^2^ Chi-square test, *MW* Mann–Whitney test

Using univariate linear regression analysis, we found that diabetes, hypertension, high baseline serum creatinine, low baseline eGFR, high levels of serum uric acid and intra-operative bleeding were the predictive factors of AKI (Table 3). Multivariate analysis of logistic regression showed that older age, smoking, high BMI, diabetes, hypertension, use of ACEI, lower preoperative haemoglobin, high baseline creatinine, low baseline eGFR, high serum uric acid level, large stone volume, large tract size, long operative time, and intra-operative bleeding were the factors predicting the occurrence of AKI (Table 4).

**Table 3:** Univariate linear regression analysis for the predictive factors of AKI

	B	*p*-value	95% CI	
Age	− 0.05	0.538	− 0.006	0.003
Gender	− 0.20	0.065	− 0.276	0.009
Smoking	− 0.06	0.124	− 0.097	0.012
BMI	0.01	0.873	− 0.01	0.011
Diabetes	**− 0.28**	** < 0.001**	**− 0.483**	**− 0.227**
Hypertension	**− 0.30**	**0.011**	**− 0.563**	**− 0.073**
ACEI	− 0.08	0.329	− 0.31	0.104
Hb	0.09	0.054	0	0.055
TLC	0.03	0.291	− 0.012	0.038
HCT	0.00	0.937	− 0.012	0.011
Baseline creatinine	**− 0.39**	**0.014**	**− 1.098**	**− 0.128**
Baseline eGFR	**− 0.34**	**0.04**	**− 0.011**	**0**
Serum uric acid	**− 0.27**	** < 0.001**	**− 0.069**	**− 0.031**
Pelvic stone	0.06	0.341	− 0.028	0.171
Upper calyx stone	0.05	0.354	− 0.046	0.129
Middle calyx stone	− 0.05	0.241	− 0.227	0.058
Lower calyx stone	0.03	0.527	− 0.053	0.102
Stone volume	0.09	0.06	− 0.003	0.148
Stone density	− 0.05	0.156	0	0
Puncture site	0.01	0.799	0	0
Number of punctures	0.06	0.16	− 0.016	0.099
Tract size	0.05	0.239	− 0.086	0.342
Operative time	− 0.01	0.865	− 0.014	0.011
Blood transfusion	− 0.09	0.14	− 0.007	0.001
Intraoperative bleeding	**− 0.16**	**0.002**	**− 0.578**	**− 0.126**

Bold indicates statistically significant difference

*B* Regression coefficient, *CI* Confidence interval

**Table 4:** Multivariate logistic regression analysis for the predictive factors of AKI

	B	SE	*p*-value	Odds	95% CI	
Age	**− 0.15**	**0.03**	** < 0.001**	**0.86**	**0.81**	**0.91**
Gender	0.46	0.43	0.281	1.59	0.69	3.68
Smoking	**− 1.93**	**0.43**	** < 0.001**	**0.15**	**0.06**	**0.34**
BMI	**− 0.57**	**0.09**	** < 0.001**	**0.57**	**0.47**	**0.68**
Diabetes	**− 5.26**	**1.07**	** < 0.001**	**0.01**	**0.00**	**0.04**
HTN	**− 3.28**	**0.54**	** < 0.001**	**0.04**	**0.01**	**0.11**
ACEI	**− 4.20**	**0.81**	** < 0.001**	**0.02**	**0.00**	**0.07**
Hb	**0.67**	**0.22**	**0.002**	**1.95**	**1.28**	**2.97**
TLC	− 0.47	0.26	0.071	0.63	0.38	1.04
HCT	0.09	0.08	0.252	1.10	0.94	1.28
Baseline creatinine	**− 13.32**	**2.33**	** < 0.001**	**0.00**	**0.00**	**0.00**
Baseline eGFR	**0.16**	**0.03**	** < 0.001**	**1.17**	**1.11**	**1.24**
Serum uric acid	**− 1.41**	**0.28**	** < 0.001**	**0.24**	**0.14**	**0.42**
Pelvic stone	− 1.95	1.04	0.06	0.14	0.02	1.09
Upper calyx stone	− 0.77	0.84	0.364	0.47	0.09	2.43
Middle calyx stone	− 0.55	0.44	0.21	0.58	0.24	1.37
Lower calyx stone	0.35	0.52	0.506	1.42	0.51	3.94
Stone volume	**0.00**	**0.00**	** < 0.001**	**1.00**	**0.99**	**1.00**
Stone density	0.00	0.00	0.931	1.00	1.00	1.00
Puncture site	− 0.07	0.41	0.868	0.93	0.42	2.09
Number of punctures	− 0.75	1.17	0.525	0.48	0.05	4.72
Tract size	**− 0.62**	**0.26**	**0.017**	**0.54**	**0.33**	**0.90**
Operative time	**− 0.11**	**0.02**	** < 0.001**	**0.89**	**0.86**	**0.93**
Blood transfusion	**− 4.70**	**1.07**	** < 0.001**	**0.01**	**0.00**	**0.08**
Intraoperative bleeding	**− 4.04**	**1.09**	** < 0.001**	**0.02**	**0.00**	**0.15**

Bold indicates statistically significant difference

*B* Regression coefficient, *SE* Standard error, *CI* Confidence interval

The adjusted Clavien-Dindo grading system was used to record postoperative complications (Table 5). Thirty-two cases developed postoperative low-grade fever, all managed by IV paracetamol. Twelve cases developed mild to moderate haematuria; eight were managed conservatively by IV fluids and haemostatic drugs, and four needed blood transfusion. Four cases complained of leakage from the site of the nephrostomy tube site after removing the ureteric catheter. Postoperative non-enhanced CT was done for these cases, and it revealed a stone in the middle third ureter in 3 cases and the lower third in one case; all were treated by ureteroscopy with double J stent insertion. Two cases developed a postoperative pneumothorax (in these cases, the supracostal puncture was done), and were managed by inserting an intercostal tube.

**Table 5:** Clavien–Dindo classification of the postoperative complications

	Calvien–Dindo	AKI (*n* = 58)		Non-AKI (*n* = 360)	
Fever	I	10	17.2%	22	6.1%
Hematuria	IIa	8	13.8%	4	1.1%
Leakage	IIIa	0	0%	4	1.1%
Pneumothorax	IIIa	0	0%	2	0.6%

The duration of hospitalization was substantially longer in patients with AKI compared to patients without AKI (3.2 days ± 0.45 vs 2.1 ± 0.42, p < 0.001).

The amount of 48-h postoperative urine output had been substantially decreased in patients with AKI compared to those without AKI [2450 (2350–2575) ml vs 5100 (4862–5250) ml, *p* < 0.001]. Postoperative serum creatinine values at 48 h follow-up were substantially higher in AKI patients than those without AKI [1.95 (1.7–2.1) vs 1.16 (1–1.26), p < 0.001] (Table 6).

**Table 6:** Comparisons between AKI and non-AKI patients regarding postoperative urine output (UOP) and serum creatinine

	AKI (*n* = 58)	Non-AKI (*n* = 360)	MW	*p*-value
24 h post-op UOP	1200 (1150–1300)	2650 (2500–2850)	1.0	< 0.001
48 h post-op UOP	2450 (2350–2575)	5100 (4862–5250)	0.0	< 0.001
24 h post-op Creat	1.98 (1.74–2.1)	1.2 (1.1–1.32)	26	< 0.001
48 h post-op Creat	1.95 (1.7–2.1)	1.16 (1–1.26)	10	< 0.001

Following 1 year of follow-up of individuals who developed AKI, we found no statistically significant variations between the preoperative and one-year postoperative values of serum creatinine and eGFR [1.52 (1.3–1.6) vs 1.48 (1.33–1.59), *p* = 0.981, 59.6 (56.5–69.5) vs 60.5 (55.2–69.1), *p* = 0.907, respectively] (Table 7). All patients with AKI were managed conservatively with no need for renal replacement treatment. In the AKI group, six patients had preoperative eGFR < 60 mL/min/1.73 m2 and continued as patients with CKD. At the end of one year of follow-up, ten new patients (17.2%) in the AKI group with preoperative eGFR > 60 mL/min/1.73 m2 developed CKD [eGFR = 49 (45.1–53.5) mL/min/1.73m2, minimum = 38.2 mL/min/1.73 m2, and maximum = 54.4 mL/min/1.73 m2), and all were managed conservatively.

**Table 7:** Serum creatinine and eGFR (baseline and 12 month post-operative) in patients with AKI

(*n* = 58)		Median (IQR)	Test	*p*-value
Serum creatinine	Baseline	1.52 (1.3–1.6)	MW = 419	0.981
	12 months	1.48 (1.33–1.59)		
eGFR	Baseline	59.6 (56.5–69.5)	MW = 413	0.907
	12 months	60.5 (55.2–69.1)		

ROC curves were constructed for the parameters, which included age, BMI, hemoglobin %, serum creatinine, eGFR, serum uric acid, and size of stone to generate a cutoff value for the significant factors identified by both univariate and multivariate analysis, with clinical and statistical significance. The cutoff value for age was > 46.5 years (79.3% sensitivity, 77.8% specificity, 78.1% PPV and 78.9% NPV, *p* < 0.001); for BMI, it was > 28.5 kg/m^2^ (75.9% sensitivity, 86.1% specificity, 84.5% PPV and 78.1% NPV, *p* < 0.001), for hemoglobin level, it was < 10.8 gm/dl (55.2% sensitivity, 78.9% specificity, 72.3% PPV and 63.8% NPV, *p* = 0.002), for serum creatinine, it was > 1.41 mg/dl (82.8% sensitivity, 89.4% specificity, 88.7% PPV and 83.8% NPV, *p* < 0.001), for eGFR, it was < 65.2 ml/min/1.73 m^2^ (85.7% sensitivity, 85% specificity, 85.1% PPV and 85.6% NPV, *p* < 0.001), for serum uric acid, it was > 5.2 mg/dl (79.3% sensitivity, 85% specificity, 84.1% PPV and 80.4% NPV, *p* < 0.001), and for the stone volume, it was > 1748 mm^3^ (68.9% sensitivity, 60% specificity, 63.3% PPV and 65.8% NPV, *p* < 0.001).

## Discussion

Although the main target in managing renal stones is to improve kidney function, this management may sometimes lead to adverse effects, which may occur at all levels of procedures from ESWL to PNL and open surgery. The prevalence of postoperative AKI following significant urological surgeries ranges from 6.7 to 38.2% [5, 9–11]. Few studies report AKI after PNL [12, 16, 17]; this may be explained as impairment of renal function without substantial perioperative problems seems minor and may pass unnoticed. AKI may increase postoperative morbidity, mortality, need for postoperative ICU admission, and demand for renal replacement treatment, so every attempt to accurately define the predictors of AKI should be made.

In the current work, we found that the incidence of AKI after PNL was 13.9%, which is consistent with the incidence described in the existing literature [5, 9–11, 18]. Age, smoking, BMI, diabetes, hypertension, use of ACEI, low preoperative haemoglobin, high baseline Creatinine, low baseline eGFR, high serum uric acid level, larger stone volume, large tract size, long operative time, and intra-operative bleeding were the predictive factors of AKI.

Renal function is affected by age as elderly patients usually suffer from associated comorbidities such as hypertension, DM, and coronary diseases; also, aging affects the cellular repairing process and decreases the ability to protect against cellular injuries, so these age-related changes may increase the susceptibility of AKI in elderly patients [19–21]. A work done by Sunil et al. demonstrated that aging is a risk indicator of AKI, which is in accordance with the current study [22].

The use of ACEI or angiotensin II inhibitors may cause renal hypoperfusion, so the use of these drugs should be considered as a risk factor for AKI. ACEI and angiotensin II receptor blockers may induce intraoperative hypotension by reducing the sensitivity to catecholamines in the renin-angiotensin system when under general anaesthesia, therefore, it is recommended to discontinue the usage of these medications on the day of the surgery [22, 23]. A work done by Sunil et al. demonstrated that the use of ACEI or angiotensin II inhibitors is a risk factor for AKI risk, which is in accordance with the current study [22].

Diabetes is recognized to possess negative impacts on the renal parenchyma and is usually associated with autonomic neuropathy that may cause some perioperative hemodynamic changes [24]. Studies performed by Sunil et al., Fayad et al., and Odzen et al. define diabetes as a risk factor for AKI [22, 25, 26].

Hypertension has a negative impact on renal function. It increases the incidence of intraoperative anaesthetic complications and intraoperative bleeding. Studies by Sunil et al. and Fayad et al. found that hypertension is a risk factor for AKI [22, 25].

This research found that low preoperative haemoglobin and high serum creatinine levels were predictive factors for postoperative AKI, especially in individuals with preoperative Hb < 10.8 g/dl and individuals who had a preoperative creatinine > 1.41 mg/dl. Research by Fulla et al., Sunil et al., and Fayad et al. defined the same factors as predictors of AKI [16, 22, 25].

Elevated levels of serum uric acid lead to the initiation of a proinflammatory process that, in turn, leads to malfunction of the endothelial cells. This dysfunction impairs autoregulation causing constriction of blood vessels in the kidneys and reduced blood flow [27, 28]. Research conducted by Yu et al. and Sunil et al. identified that elevated levels of serum uric acid are predictors of AKI [12, 22].

Multiple punctures and a larger tract size result in substantial nephron injury, increase the operative time and the possibility for perioperative complications and were identified as risk factors of AKI [17, 29]. However, we did not find the number of tracts to be an independent risk factor in this work, as all our cases were done through 1–2 punctures.

Stone factors such as large stones and stones with high density increase the complexity and the operative time of the procedure, with the increased possibility of perioperative blood loss and infectious complications leading to a higher risk of AKI. Multiple previous studies defined these stone factors as predictors of AKI [16, 17, 30].

Intraoperative hypotension (defined as mean arterial blood pressure of less than 70mm Hg for more than 5 min), can cause about 2/3 of cases of acute tubular necrosis and may lead to postoperative AKI. This may explain why intraoperative blood loss, perioperative blood transfusion or low preoperative haemoglobin levels are risk factors for AKI [31]. A work by Yu et al. detected that intraoperative hypotension was a major risk factors for AKI [12].

AKI results in an extended duration of hospitalization. In our work, the mean time of hospital stay was significantly longer in patients with AKI than those without AKI (3.2 days ± 0.45 vs 2.1 ± 0.42, *p* < 0.001).

### Limitations of the study

This work had some limitations including relatively small number of patients, short follow-up period, and proteinuria, as a KDIGO criterion for CKD, was not assessed.

## Conclusions

In the current study, we found that age, smoking, BMI, diabetes, hypertension, use of ACEI, low preoperative haemoglobin, high baseline creatinine, low baseline eGFR, high uric acid level, large stone volume, large tract size, long operative time, and intra-operative bleeding were the predictive factors of AKI. 13.9% of our cohort patients developed AKI, so identification and optimization of these risk factors and meticulous technique during the PNL procedures have to be taken into consideration to avoid and lower the incidence of postoperative AKI.

## Data Availability

All available data were included in the figures and tables.
